# Double-Stranded RNA Technology to Control Insect Pests: Current Status and Challenges

**DOI:** 10.3389/fpls.2020.00451

**Published:** 2020-04-21

**Authors:** Olivier Christiaens, Steve Whyard, Ana M. Vélez, Guy Smagghe

**Affiliations:** ^1^Department of Plants and Crops, Ghent University, Ghent, Belgium; ^2^Department of Biological Sciences, University of Manitoba, Winnipeg, MB, Canada; ^3^Department of Entomology, University of Nebraska-Lincoln, Lincoln, NE, United States

**Keywords:** RNA interference, RNAi, pest management, insect pests, dsRNA, host-induced gene silencing (HIGS), spray-induced gene silencing (SIGS), virus-induced gene silencing (VIGS)

## Abstract

Exploiting the RNA interference (RNAi) gene mechanism to silence essential genes in pest insects, leading to toxic effects, has surfaced as a promising new control strategy in the past decade. While the first commercial RNAi-based products are currently coming to market, the application against a wide range of insect species is still hindered by a number of challenges. In this review, we discuss the current status of these RNAi-based products and the different delivery strategies by which insects can be targeted by the RNAi-triggering double-stranded RNA (dsRNA) molecules. Furthermore, this review also addresses a number of physiological and cellular barriers, which can lead to decreased RNAi efficacy in insects. Finally, novel non-transgenic delivery technologies, such as polymer or liposomic nanoparticles, peptide-based delivery vehicles and viral-like particles, are also discussed, as these could overcome these barriers and lead to effective RNAi-based pest control.

## Introduction

Insects are our most serious competitors for food and fiber and are vectors of some of our most serious diseases. Chemical pesticides are routinely used to protect crops and to reduce the spread of insect-borne diseases. Due to their frequent use, there are increasing incidences of insecticide resistance to many of the most commonly used insecticides (Sparks and Nauen, 2015). In addition, there is increasing public concern over the risk that many of these chemicals pose to the environment and to human and livestock health (Damalas and Eleftherohorinos, 2011; Nicolopoulou-Stamati et al., 2016). Together, these issues provide compelling reasons to find safer, more pest-specific alternatives to control pest insects. One technology that offers the promise of a reduced risk approach to insect pest control is RNA interference (RNAi). RNAi is a sequence-specific method of suppressing a targeted gene’s expression, and because each species is defined by the uniqueness of its genes’ sequences, RNAi can potentially be designed in a species-specific manner. By targeting genes essential for pest insect’s growth, development, or reproduction, RNAi could be used selectively to kill pest insects without adversely affecting non-target species (Whyard et al., 2009).

RNAi is a naturally occurring cellular defense system mediated by double-stranded RNA (dsRNA). In most eukaryotes, long dsRNA found within a cell is seen as either a source of viral infection or as evidence of transposon activity, both of which the cell will seek to suppress (Obbard et al., 2008). The first component of the RNAi machinery to respond to the dsRNA is the RNase III endonuclease Dicer-2 (Dcr-2), which cleaves the dsRNA into short (typically 19-21 nt long) interfering RNAs (siRNAs). Dicer-2, with the help of dsRNA-binding proteins such as R2D2, facilitates the transfer of the siRNA to the RNA−induced silencing complex (RISC). Within RISC, the siRNA is unwound, and one strand, the passenger strand, is eliminated. Using the retained guide strand, the activated RISC complex scans cellular mRNAs, and an Argonaute protein (Ago2) within RISC cleaves transcripts with complementarity to the siRNA, thus silencing gene expression (Okamura et al., 2004).

Due largely to this sequence specificity, growing numbers of research groups and biotechnology industries are exploring the efficacy of using dsRNA as a new source of environmentally friendly, potentially species-specific insecticides. Some insects, particularly of the order Coleoptera (beetles), have proven highly susceptible to dsRNA (Baum and Roberts, 2014), such that only small quantities of ingested dsRNA can induce RNAi, causing both transcript knockdown, and where essential genes were targeted, insect mortality. A particularly intriguing aspect of RNAi is that in these highly susceptible insects, the dsRNA is not only capable of entering gut cells, but can spread to other tissues to induce systemic RNAi (Joga et al., 2016). The systemic nature of RNAi is particularly useful in the development of a broader range of potential insecticidal dsRNAs that can target essential genes in many other tissues of the pest insects (Huvenne and Smagghe, 2010).

Not all insects, however, respond equally well to ingested dsRNA. Insects of the order Lepidoptera (moths and butterflies), Diptera (flies and mosquitoes), and Hemiptera (aphids, hoppers, stinkbugs), respond to dsRNA with greater variability than that seen in beetles (Cooper et al., 2019). If RNAi is to be developed for insecticidal applications in a broader range of insects, it is important that we understand some of the barriers to efficient RNAi, and consider how we might deliver dsRNA to different insects to maximize the potential of RNAi for insect control more fully. In this review, we will explore the potential for dsRNA-based insecticides by considering the methods that have been used to date to deliver dsRNA, what barriers can limit RNAi efficiency in some insects, and how alternative delivery methods may help overcome some of the limitations in certain insects.

## Application of RNAi in the Field

Application of RNAi in agriculture, more specifically in pest or pathogen control, can be achieved in different ways, namely by host-induced gene silencing (HIGS), spray-induced gene silencing (SIGS) or virus-induced gene silencing (VIGS).

HIGS entails the creation of transgenic crops that express the dsRNA specific for the pest or pathogen. The first commercial RNAi product targeting an insect pest is a transgenic corn crop, developed by Monsanto (currently Bayer CropScience), which expresses a hairpin dsRNA targeting the *snf7* gene in the Western corn rootworm, *Diabrotica virgifera virgifera* (Bolognesi et al., 2012; Bachman et al., 2013). This new RNAi construct is also stacked with two *Bacillus thuringiensis* Cry proteins (Cry3Bb1 and Cry34/35Ab), in an effort to delay the evolution of resistance (Head et al., 2017). This product will be marketed under the trade name of SmartStax Pro, was approved in 2017 by the United States Environmental Protection Agency (EPA, 2017), and is expected to be released for commercial use by the end of the decade. SmartStax Pro is considered a milestone in the use of RNAi technology in agriculture (Head et al., 2017).

Other genes have also demonstrated plant protection against *D. v. virgifera*, including the vacuolar proton pump, *V-ATPase A* (Baum et al., 2007), the septate junction proteins *snakeskin* (*ssj1*) and *mesh* (*ssj2*) (Hu et al., 2016), *Troponin I* (Fishilevich et al., 2019), SNARE binding protein *Ras opposite/Sec1*, RNA polymerase II subunit *RpII140*, FACT complex protein dre4/spt16 (Knorr et al., 2018), and *Sec23* subunit of the coat protein complex II (COPII) (Vélez et al., 2019). HIGS in other insects has been explored with a high degree of variability in the response (Yu et al., 2016; Zhang et al., 2017).

VIGS is a rather novel delivery method that is based on viruses engineered to produce the desired dsRNA in the pest itself (Kolliopoulou et al., 2017). For example, an insect virus could be modified to contain an insect-specific sequence in its genome, homologous to an insect’s essential gene. Infection and replication of the virus would then lead to the production of dsRNA molecules directly in the insect cells. A major advantage of this delivery method is that a very high efficiency can be achieved, even in otherwise recalcitrant cells. Relying on the virus’s own infection processes, physiological and cellular barriers for the uptake of dsRNA from the environment are thus bypassed. Furthermore, viruses can be very host-specific, thereby providing another layer of species-specificity to this technology. A proof-of-concept of VIGS directed against insects was recently provided by Taning et al. (2018), who successfully modified Flock house virus (FHV) to express *Drosophila melanogaster*-specific dsRNA.

A VIGS-like technology has also been proposed using various microbes, such as bacteria, yeast, or fungi that are engineered to serve as vectors for gene-silencing induction through the continuous production of si/dsRNA into the host (Whitten et al., 2016). A review of the use of bacteria and viruses for dsRNA delivery is provided in Joga et al. (2016) and Zotti et al. (2018). The potential, successes and concerns on micro-organisms or derived products as delivery methods for insect and disease management, are discussed in more detail in a later section.

Finally, many efforts have also focused on the use of non-transgenic, spray-based pesticidal dsRNAs (SIGS) to control pests and pathogens. SIGS can also be used for root absorption and trunk injections, where insects can acquire dsRNA through sucking and chewing, a review of this delivery method is provided in Joga et al. (2016) and Zotti et al. (2018). Given the low persistence of dsRNA molecules in the environment, SIGS will most likely need special formulations to increase the stability, and if possible, also increase the RNAi efficacy in the insect. Furthermore, the exposure of target pests through SIGS is likely to be lower compared to transgenic plants, since plants offer the possibility of continuous high expression of the insecticidal dsRNA. Therefore, spray-based applications might only become a reality for those insects that are more sensitive to dietary uptake of dsRNA.

In the following sections, the variation in RNAi responses between insects will be discussed, focusing on physiological and cellular barriers that affect RNAi efficacy. In the last section, we will focus on formulations and delivery methods that could improve non-transgenic spray-based RNAi approaches and eventually perhaps lead to effective and sustainable RNAi-based control strategies against pests and pathogens.

## Variation in RNAi Response Between Insects

The ability of insects to acquire dsRNA through feeding (i.e., environmental RNAi) will determine the potential use of RNAi technology for insect pest management. However, different insect orders respond differently to dsRNA. From the various insects studied to date, coleopterans are, in general, highly sensitive to RNAi, while Hemiptera, Orthoptera, Diptera, Hymenoptera, and Lepidoptera have different levels of variability in their responses (Table 1). Multiple mechanisms appear to affect the efficiency of RNAi in different insect species, including: (1) instability of dsRNA before and after it enters the insect; (2) insufficient dsRNA internalization; (3) deficient RNAi machinery; (4) impaired systemic spreading; and (5) refractory gene targets. Cooper et al. (2019) provide an extensive review of this topic. There are not only differences in the responses across orders, but also within species, life stages, tissues, and genes (Terenius et al., 2011; Wynant et al., 2012; Guo et al., 2015; Pereira et al., 2016; Singh et al., 2017; Vogel et al., 2018; Cooper et al., 2019; Grover et al., 2019).

**TABLE 1:** Mechanisms of dsRNA cellular uptake identified in different insect species.

Order	Species	Environmental RNAi	Sid-1	Endocytosis	References
Diptera	*Drosophila melanogaster*	+	No	Yes	Saleh et al., 2006
	*Bactrocera dorsalis*	+	No	Yes	Li X. X. et al., 2015
Coleoptera	*Tribolium castaneum*	+	No	Yes	Tomoyasu et al., 2008; Xiao et al., 2015
	*Diabrotica virgifera virgifera*	+ +	Yes	Yes	Miyata et al., 2014; Pinheiro et al., 2018
	*Leptinotarsa decemlineata*	+ +	Yes	Yes	Cappelle et al., 2016
Lepidoptera	*Spodoptera frugiperda*	+ but no endosomal release	Not determined	Yes	Yoon et al., 2017
	*Bombyx mori*	–	No	Not determined	Tomoyasu et al., 2008
Orthoptera	*Schistocerca gregaria*	–	No	Yes	Wynant et al., 2014
	*Locusta migratoria*	–	No	Not determined	Luo et al., 2012
Hymenoptera	*Apis mellifera*	+	Yes	Not determined	Aronstein et al., 2006
Hemiptera	*Nilaparvata lugens*	–	Yes	Not determined	Xu et al., 2013

*Adapted from Cappelle et al. (2016). RNAi: ++, present and robust; +, present but not robust; –, not present.*

**FIGURE 1:**
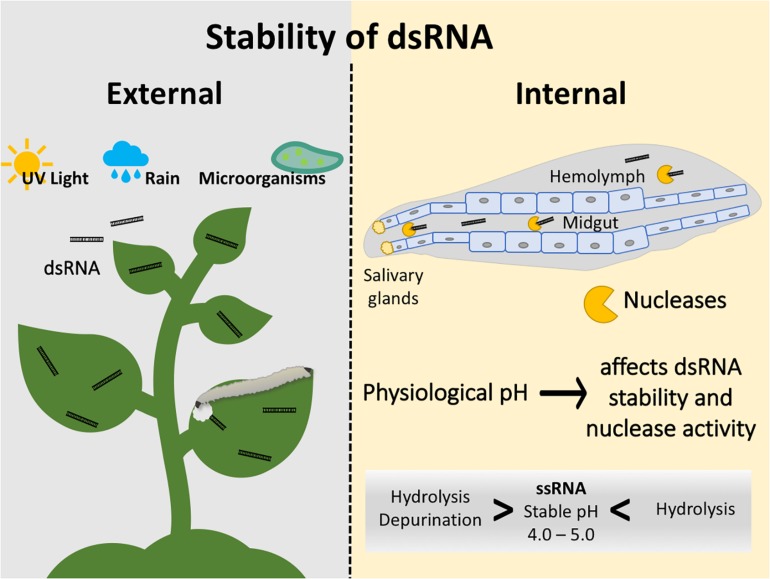
Factors affecting the stability of dsRNA in the environment and inside the insect. External factors include degradation by UV light and microorganisms and runoff of sprayable dsRNAs by rain. Internal factors include nucleases present in salivary glands, midgut, and hemolymph. Physiological pH affects dsRNA stability and nuclease activity; ssRNA is stable at a pH of 4.0–5.0.

As noted earlier, the delivery of dsRNA for insect pest management could be through expression in transformed plants, microbes or delivery as a spray-based insecticidal dsRNA. Regardless of the delivery mechanisms, the dsRNA must be stable before it is consumed by the insect to generate an effect. For spray-based insecticides, factors such as UV light and microorganisms can degrade naked dsRNA in the environment. Whereas rain can hydrate dsRNA, making it less stable (Figure 1). In the next section, the strategies used to overcome these issues are described. The dsRNA may not only be destabilized by environmental factors, but its availability to feeding insects could also be impaired by binding to environmental molecules that interfere with cellular uptake. For example, in honey bee larvae, RNAi efficacy was reduced as the dsRNA was bound to the main ingredient of larval diet, royal jelly. Furthermore, when *D. v. virgifera* adults were fed with an artificial diet treated with royal jelly containing a lethal concentration of *D. v. virgifera vATPase-A* dsRNA, no mortality was observed (Vélez et al., 2016).

Once the insect has consumed the dsRNA, the dsRNA must avoid the degradation by nucleases from salivary glands, midgut, and hemolymph of the insect (Figure 1). Studies with hemipterans, including the tarnished plant bug, *Lygus lineolaris*, and the peach aphid, *Acyrthosiphon pisum*, have shown that dsRNA is degraded by saliva (Allen and Walker, 2012; Christiaens et al., 2014). Similarly, research performed with the tobacco hornworm, *Manduca sexta*, and the German cockroach, *Blatella germanica*, demonstrated that dsRNA degraded in the hemolymph after 1 and 24 h, respectively (Garbutt et al., 2013). Studies performed with the silkworm, *Bombyx mori*, the desert locust, *Schistocerca gregaria*, and the Colorado potato beetle, *Leptinotarsa decemlineata*, also demonstrated that midgut juices degrade dsRNA (Liu et al., 2013; Spit et al., 2017). In *B. mori*, dsRNA degraded within only ten minutes of exposure to midgut nucleases (Liu et al., 2013). The efficiency of nucleases within insect guts can vary from one species to the next. For example, 10-minute *in vitro* incubations of dsRNA with serial dilutions of gut juices showed that dsRNA disappeared much faster in *S. gregaria* compared to *L. decemlineata.* Similarly, a comparative study between two weevil species belonging to the genus Cylas, indicated that dsRNA degradation in the gut could be a source of variability, even between two very closely related species (Christiaens et al., 2016; Prentice et al., 2017). Furthermore, a study demonstrated that *L. decemlineata* with knockdown of nucleases incur less damage on potato plants expressing dsRNA (Spit et al., 2017), similar findings were observed in the sweetpotato weevil *Cylas puncticollis* (Prentice et al., 2019). These studies suggest that combining the knockdown of nucleases and a lethal gene can improve the use of RNAi as a strategy for plant protection. The variability in the stability of dsRNA in different parts of the insect body (e.g., midgut vs. hemolymph), could also be explained by differences in physiological pH that could affect dsRNA stability and nucleases’ enzymatic activity. ssRNA is most stable at pH 4.0–5.0, while it is susceptible to hydrolysis at pH > 6.0 and <2.0, and to depurination at <3.0 (Figure 1; Cooper et al., 2019). However, no experimental evidence is available so far to determine the effect of physiological pH on dsRNA stability and the activity of nucleases.

**FIGURE 2:**
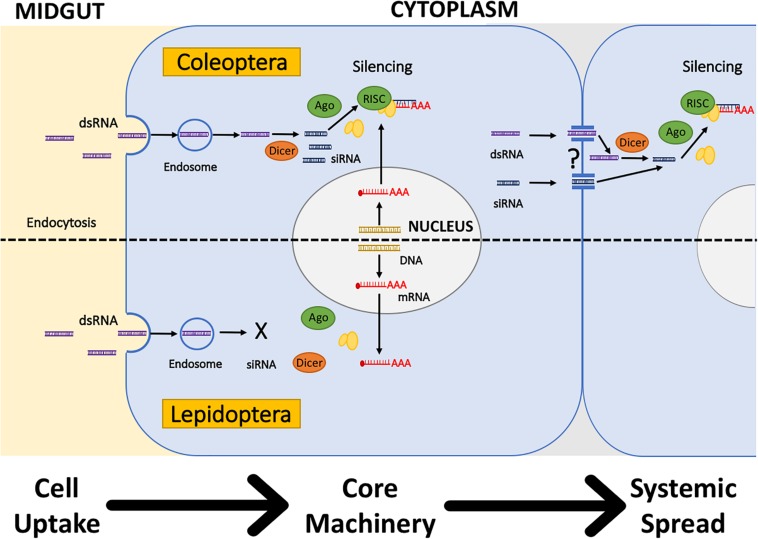
Hypothetical differences in the cellular internalization, processing, and systemic spread of dsRNA in Coleoptera **(Top)** and Lepidoptera **(Bottom)**. *Cell Uptake and Processing*: Clathrin-dependent endocytosis is hypothesized to be the primary dsRNA uptake mechanism in insects. In Coleoptera, dsRNA is released from the endosome and processed by the core RNAi machinery to silence mRNA. In Lepidoptera, dsRNA is not released from the endosome, avoiding dsRNA contact with the core RNAi machinery. *Systemic Spread*: In Coleoptera, experimental evidence suggests that systemic spread occurs, but is not clear if it is in the form of dsRNA or siRNA. In Lepidoptera, no current evidence exists regarding the systemic spread. Adapted from Shukla et al. (2016).

After the dsRNA has overcome the initial barriers of dsRNA degradation in the environment, external and internal, the next barrier is the internalization of the dsRNA in the cell (Figure 2). Two mechanisms of cellular uptake of dsRNA have been identified in insects: Sid-like transmembrane channels, and clathrin-dependent endocytosis (Table 1). The role of Sid-like transmembrane channels dsRNA uptake was first described in the nematode *Caenorhabditis elegans* (Winston et al., 2007; Whangbo and Hunter, 2008). In insects, Sid-like genes have been identified in Coleoptera, Hemiptera, and Lepidoptera, but the role in cellular uptake has not been directly evidenced to date (Tomoyasu et al., 2008; Xu et al., 2013; Cappelle et al., 2016; Pinheiro et al., 2018). Whereas, clathrin-dependent endocytosis seems to play the primary role in the uptake of dsRNA in multiple insects (Saleh et al., 2006; Xiao et al., 2015; Cappelle et al., 2016; Pinheiro et al., 2018). Other mechanisms involved in dsRNA/siRNA uptake in mammals such as caveolar endocytosis and micropinocytosis remain unexplored in insects. Vélez and Fishilevich (2018) provide a review of the evidence that supports the key role of endocytosis in the uptake of dsRNA and discusses the role of other components of the cellular membrane transport in the efficiency of RNAi.

Uptake of dsRNA is also affected by the dsRNA length and structure, and the vehicle used to deliver the dsRNA. For example, in *D. v. virgifera*, uptake of naked dsRNA is limited to long dsRNA, no shorter than 60 bp (Bolognesi et al., 2012; Li H. et al., 2015). Several chemical modifications of dsRNA and vehicles of delivery are discussed in the next section. Once the dsRNA enters the cell through endocytosis, the dsRNA needs to be released from the endosome to get in contact with the RNAi machinery (i.e., *dcr-2* and RISC) and generate knockdown of the targeted gene (Saleh et al., 2006; Xiao et al., 2015). Endosomal release occurs after the endosome is acidified. Research performed with the fall armyworm, *Spodoptera frugiperda*, demonstrated that the lack of endosomal release of the dsRNA leads to low sensitivity to RNAi in Lepidoptera (Figure 2; Shukla et al., 2016; Yoon et al., 2017). Another example of the potential limitation of uptake in RNAi efficiency is the identification of *D. v. virgifera* resistant to *snf7* dsRNA. Resistance to *snf7* dsRNA showed cross-resistance to other dsRNAs, and microscopy experiments determined that resistance was linked to the uptake of dsRNA (Khajuria et al., 2018).

After the release of the dsRNA from the endosomes, the dsRNA is processed by the RNAi (core) machinery to generate sequence-specific gene knockdown (Okamura et al., 2004). In eukaryotes, three RNAi pathways have been described: (1) siRNA consisting of an exogenous and endogenous pathway for viral and transposon defense, respectively; (2) microRNA (miRNA) a pathway that regulates gene expression at the transcription level, and (3) piwi-interacting RNA (piRNA) which functions in the epigenetic control of genomic elements (Kingsolver et al., 2013). While the RNAi mechanism is conserved across eukaryotes, differences in the proteins involved in the core machinery of the three different pathways vary between clades. Plants have four Dicer-like proteins, while insects have two, and annelids, nematodes, mollusks, and higher animals only have one (Mukherjee et al., 2013). Ago-like proteins are even more diverse, with insects having four (Ago1, Ago2, Ago3, Piwi, and Aubergine), humans have eight, and *Arabidopsis thaliana* plants have ten (Hock and Meister, 2008). In insects, the different pathways involve different proteins, including different Dicer, Ago, and other ancillary proteins (Cooper et al., 2019). When thinking about RNAi efficiency, it is useful to think about the duplication of core RNAi pathway genes (Tomoyasu et al., 2008; Guo et al., 2015). Yoon et al. (2016) demonstrated that *ago1*, *ago2*, and *aubergine* were essential for RNAi in *L. decemlineata* cell line. Interestingly, *ago1* and *aubergine* are part of the miRNA and piRNA pathways, respectively. Other components of the miRNA pathway also seemed to play a partial role in the siRNA pathway. The results of this study suggest that gene duplication might explain the effectiveness of RNAi in Coleoptera. However, the involvement of miRNA and piRNA in dsRNA-mediated RNAi needs to be further investigated in Coleoptera and other insects (Yoon et al., 2016).

In addition to gene duplication, the baseline mRNA expression of core machinery genes could also explain the differences in the RNAi efficiency between different insect orders. To test this hypothesis, Davis-Vogel et al. (2018) evaluated eight proteins from the siRNA and miRNA pathways among three agricultural pests from three different orders: *D. v. virgifera* (Coleoptera), *S. frugiperda* (Lepidoptera), and *Nezara viridula* (Hemiptera). In this study, researchers compared transcript levels of core machinery proteins Drosha, Dcr-1, Dcr-2, Pasha, Loquacious, R2D2, Ago-1, and Ago2 among the three species. Direct comparison of the proteins in the three insects revealed that *D. v. virgifera* had an increase in *loquacious* expression, an insect with a robust RNAi response (Davis-Vogel et al., 2018). In a different study, low *r2d2* gene expression was suggested as one of the reasons for a *B. mori* ovarian cell line insensitivity to RNAi (Swevers et al., 2011). These studies suggest that differential gene expression of core machinery genes might influence the RNAi response in insects, but further evidence is needed.

Maximizing the utility of RNAi in insects requires the systemic spread of the RNAi response throughout the insect body. A strong systemic response requires a sufficient number of siRNAs to reach a high number of cells in the insect body. In *C. elegans*, the RNA-dependent RNA Polymerase (RdRP) generates secondary siRNAs from the primary siRNA (Sijen et al., 2001). However, RdRP in arthropods is restricted to the tick lineage and is not found in insects (Gordon and Waterhouse, 2007). In insects, evidence of a systemic RNAi response has only been indirectly determined by observing gene knockdown in tissues distant from the place of uptake (i.e., hemolymph or gut) (Bolognesi et al., 2012; Ivashuta et al., 2015; Khajuria et al., 2015; Niu et al., 2017; Li et al., 2018). Only one study in *D. v. virgifera* has shown the spread of the RNAi response using microscopy. Researchers reported the reduction of mRNA molecules in gut and fat body, but there was no detection of secondary siRNA production, suggesting that the origin of siRNAs is restricted to the processing of the initial dose of dsRNA (Li et al., 2018). Even though systemic RNAi is observed in insects, the specific mechanisms, genes involved in the spread of the dsRNA, and the form of the signal (dsRNA or siRNA) are yet to be unraveled (Vélez and Fishilevich, 2018). Two mechanisms of transport of dsRNA between cells have been suggested in the context of viral infection: (1) *via* derived complementary viral DNAs (vDNA) used as template for *de novo* synthesis of secondary viral siRNAs (vsRNAs) released in exosomes (Tassetto et al., 2017); and (2) through nanotube-like structures observed in *D. melanogaster* cultured cells (Karlikow et al., 2016). Further research on systemic RNAi will provide insights to improve RNAi use in pest management in other insect orders.

Finally, another factor that has been described to interfere with RNAi efficiency is the presence of viruses in the targeted insect. Since the RNAi pathway is an antiviral defense mechanism, viruses can influence the core machinery availability (Christiaens and Smagghe, 2014). Furthermore, since viruses have evolved with the RNAi defenses, some of them have developed mechanisms to inhibit the RNAi proteins (Haasnoot et al., 2007). For example, in *Drosophila*, a protein from the Flock House virus binds to the dsRNA, which in turn cannot be diced by Dicer and this affects binding to the RISC complex (Chao et al., 2005). While in honey bees, injection of *GFP* dsRNA and Sindbis virus regardless of the sequence, reduced virus infection (Flenniken and Andino, 2013). Swevers et al. (2013) provide a review of the impact of virus infection on the RNAi machinery in insects.

## Methods of Delivery and Formulations

Many efforts have been made to overcome these physiological and cellular barriers in different insect species and increase RNAi efficacy in insects for non-transgenic, SIGS. These efforts range from chemical modifications of the dsRNA molecule to the use of a variety of delivery vehicles and other formulations. Recently, a study reported that the addition of EDTA as a co-formulant could increase RNAi efficacy in the Neotropical stinkbug *Euschistus heros*. First, they demonstrated *in vitro* that the addition of EDTA, which is a known inhibitor of metalloenzymes, led to increased stability of the dsRNA in *E. heros* saliva. They also observed a significant increase in RNAi-induced mortality for one of the two tested target genes (Castellanos et al., 2019).

Chemical modifications to the dsRNA (or siRNA) could also improve its stability in different environments. For example, the use of siRNAs that were modified to contain two 2’−methoxyl−nucleotides on each end of the siRNAs led to effective RNAi silencing in the diamondback moth, *Plutella xylostella* (Gong et al., 2011, 2013). Literature from the vertebrate RNAi field also suggests that chemical modifications could reduce the potential of off-target effects when using short siRNAs (Jackson et al., 2006). Several smaller industry players are now investigating the potential of chemically modified dsRNA or siRNA for pest control.

DsRNA could also be delivered by micro-organisms in order to overcome or bypass the RNAi-barriers in insects. For example, RNAi can be achieved by feeding insects with dsRNA-producing *E. coli* (Joga et al., 2016; Vatanparast and Kim, 2017). Feeding insects with dsRNA-producing bacteria could lead to a more sustained release of the dsRNA in the insect and could help avoid rapid degradation in the digestive system. RNAi efficiency and its use for pest control could even be increased further by using engineered symbionts of the target pest. Whitten et al. (2016) engineered such symbionts for two insect pests: the Western flower thrips *Frankliniella occidentalis* and the kissing bug *Rhodnius prolixus*. In both cases, a long-lasting RNAi silencing effect was observed, which was a considerable improvement compared to other feeding or injection delivery methods. Furthermore, it was observed that the symbiont was also horizontally transmitted through the population *via* feces (Whitten et al., 2016; Whitten and Dyson, 2017).

Another way to overcome some of the barriers is by using nanocarriers that could increase the stability of dsRNA in the insect body or increase cellular uptake rate of dsRNA upon ingestion. Examples of these are liposomes, polymers, and peptides. In one of the earliest studies on the potential of exogenous insecticidal dsRNA, Whyard et al. (2009) demonstrated that feeding Lipofectamine-encapsulated dsRNA targeting essential genes could lead to an efficient gene silencing and high mortality in the fruit fly *D. melanogaster*, while naked dsRNA had no observable effect. This was later confirmed by Taning et al. (2016) in the pest fruit fly *Drosophila suzukii*, suggesting that fruit flies have an impaired cellular uptake capacity for dsRNA. Lipofectamine or other liposomic compounds have also proven their ability to improve RNAi efficacy in other insects, such as the hemipteran stinkbug *E. heros* (Castellanos et al., 2019), the cockroach *B. germanica* (Huang et al., 2018) and the tick *Rhipicephalus haemaphysaloides* (Zhang et al., 2018). Another intriguing concept is the use of so-called bacterial minicells. Although research on these vesicles for RNAi applications is scarce, certain startup companies, e.g., Agrospheres, are exploring such technology for dsRNA or siRNA delivery in the field.

Another class of promising compounds is cationic polymers. These polymers could be specifically synthesized to protect dsRNA against nucleolytic degradation at various pH conditions and could also improve cellular uptake. An early example of this was the use of the natural polymer chitosan to improve RNAi efficacy in mosquitoes (Zhang et al., 2010). Since then, many other studies have proven the potential of these carriers in other species, including *Spodoptera exigua* (Christiaens et al., 2018), *Ostrinia furnacalis* (He et al., 2013), *S. frugiperda* (Parsons et al., 2018), and *Aedes aegypti* (Lopez et al., 2019). Recently, a guanylated polymer developed at Ghent University, Belgium, was able to protect dsRNA against nucleolytic degradation in a high alkali environment and significantly improve RNAi efficacy in the lepidopteran *S. exigua* (Christiaens et al., 2018). Additionally, the polymer appeared to also improve cellular uptake of the dsRNA in lepidopteran midgut cells. While the underlying mechanism is unknown, the polymer may bypass the typical endocytic pathways known to be involved in cellular dsRNA uptake (Christiaens et al., 2018).

Recently, a non-toxic and biodegradable layered-double-hydroxide nanoparticle, called BioClay, was developed at the University of Queensland, Australia (Mitter et al., 2017). This nanoparticle could be loaded with dsRNA and leads to a sustained release, as the BioClay degrades. In their study, they opted for the delivery of a plant virus targeting dsRNA and were able to detect this dsRNA for at least 30 days after being sprayed on the plants, which was a considerable improvement compared to naked dsRNA. Functionally, it led to a successful antiviral effect in the plant for at least 20 days, which suggests that the dsRNA, either with or without the nanoparticle, is being taken up by the plant cells (Mitter et al., 2017).

Peptide- or protein-based nanoparticles could also be used as a delivery vehicle. Recently, cell-penetrating peptides (CPP) were used for the first time as a carrier for dsRNA in insects. Gillet et al. (2017) synthesized a recombinant fusion protein containing a CPP amino acid sequence fused to a dsRNA binding domain. Nanoparticles comprising a dsRNA-peptide complex significantly improved RNAi efficacy in the RNAi-insensitive cotton boll weevil *Anthonomus grandis*. This promising result should encourage the development and testing of other types of peptides or proteins for their applicability to other pest insect control systems.

Finally, one potential delivery method that has shown promise in vertebrate systems, but has not been explored in insects, is viral-like particles (VLPs). VLPs can be produced in micro-organisms and have the ability to self-assemble *in vitro*, allowing the integration of the dsRNA inside the particle (Hoffmann et al., 2016). Alternatively, the dsRNA and VLPs could also be co-expressed in bacteria, allowing immediate use in the field or purification of the dsRNA-containing particles. The advantages are similar to the use of replicating engineered viruses, in that they could allow efficient cellular uptake and protection of the dsRNA in the extracellular environments of the insect. Furthermore, they might also be able to offer a certain degree of host specificity. VLPs could be a more realistic alternative to the use of engineered viruses, since they would not have some of the biosafety or public acceptance concerns that are associated with the release of genetically modified viruses.

Further inspiration for novel dsRNA delivery methods could also be taken from the medical field, where pharmaceutical Research and Development has been searching for ways to overcome similar barriers in vertebrates. Of course, such formulations could also have an impact on food/feed safety risk assessment of these RNAi-based pest control products, so these will have to be taken into account during the risk assessment process.

## Conclusion

RNAi continues to be considered a promising pest management strategy, largely due to its potential for environmentally friendly control. The first RNAi-based products, targeting insects that are highly sensitive to dietary uptake of dsRNA, will soon be commercially available. However, the application against a wide range of insect species is still hindered by a number of challenges. These challenges, which are largely linked to the variable RNAi sensitivity of oral RNAi in insects, are likely to be addressed by the use of different formulation strategies improving dsRNA persistence and cellular uptake in these insects. Certain proof-of-concept studies in this field have been published already and show promise, but further progress needs to be made before these RNAi products against a wide range of insect species can compete with the currently used conventional chemical pesticides. Research on the effect of nucleases and physiological pH in dsRNA stability, mechanisms of dsRNA uptake and systemic spread, interaction with viruses, and potential mechanisms of resistance will aid in improving this technology in the future.

## Author Contributions

OC, SW, AV, and GS conceived the idea, wrote, and approved the final manuscript.

## Disclaimer

The opinions expressed and arguments employed in this paper are the sole responsibility of the authors and do not necessarily reflect those of the OECD or of the governments of its Member countries.

## Conflict of Interest

The authors declare that the research was conducted in the absence of any commercial or financial relationships that could be construed as a potential conflict of interest.
